# Mapping QTL conferring resistance in maize to gray leaf spot disease caused by *Cercospora zeina*

**DOI:** 10.1186/1471-2156-15-60

**Published:** 2014-05-22

**Authors:** Dave K Berger, Maryke Carstens, Jeanne N Korsman, Felix Middleton, Frederik J Kloppers, Pangirayi Tongoona, Alexander A Myburg

**Affiliations:** 1 Department of Plant Science, Forestry and Agricultural Biotechnology Institute (FABI), Plant Sciences Complex, University of Pretoria, Private Bag X20, Hatfield 0028, South Africa; 2 PANNAR SEED (Pty) Ltd, PO Box 19, Greytown, South Africa; 3 African Centre for Crop Improvement, University of KwaZulu-Natal, Pietermaritzburg, South Africa; 4 Department of Genetics, Forestry and Agricultural Biotechnology Institute (FABI), University of Pretoria, Pretoria, South Africa

**Keywords:** Gray leaf spot, Grey leaf spot, GLS, *Cercospora*, QTL, Maize, Corn

## Abstract

**Background:**

Gray leaf spot (GLS) is a globally important foliar disease of maize. *Cercospora zeina*, one of the two fungal species that cause the disease, is prevalent in southern Africa, China, Brazil and the eastern corn belt of the USA. Identification of QTL for GLS resistance in subtropical germplasm is important to support breeding programmes in developing countries where *C. zeina* limits production of this staple food crop.

**Results:**

A maize RIL population (F7:S6) from a cross between CML444 and SC Malawi was field-tested under GLS disease pressure at five field sites over three seasons in KwaZulu-Natal, South Africa. Thirty QTL identified from eleven field trials (environments) were consolidated to seven QTL for GLS resistance based on their expression in at least two environments and location in the same core maize bins. Four GLS resistance alleles were derived from the more resistant parent CML444 (bin 1.10, 4.08, 9.04/9.05, 10.06/10.07), whereas the remainder were from SC Malawi (bin 6.06/6.07, 7.02/7.03, 9.06). QTLs in bin 4.08 and bin 6.06/6.07 were also detected as joint QTLs, each explained more than 11% of the phenotypic variation, and were identified in four and seven environments, respectively. Common markers were used to allocate GLS QTL from eleven previous studies to bins on the IBM2005 map, and GLS QTL “hotspots” were noted. Bin 4.08 and 7.02/7.03 GLS QTL from this study overlapped with hotspots, whereas the bin 6.06/6.07 and bin 9.06 QTLs appeared to be unique. QTL for flowering time (bin 1.07, 4.09) in this population did not correspond to QTL for GLS resistance.

**Conclusions:**

QTL mapping of a RIL population from the subtropical maize parents CML444 and SC Malawi identified seven QTL for resistance to gray leaf spot disease caused by *C. zeina*. These QTL together with QTL from eleven studies were allocated to bins on the IBM2005 map to provide a basis for comparison. Hotspots of GLS QTL were identified on chromosomes one, two, four, five and seven, with QTL in the current study overlapping with two of these. Two QTL from this study did not overlap with previously reported QTL.

## Background

Gray leaf spot (GLS) is a foliar disease of maize that was highlighted as a threat to maize production in the USA in the 1980s [1], reported in South Africa in the 1990s [2], and currently has a worldwide distribution in maize production areas, including South America [3] and China [4]. One of the main reasons for emergence of the disease in commercial settings is the increasing use of conservation tillage, which allows fungal inoculum to build up on crop residues [5]. Adjacent or subsequent maize crops are then readily infected. Considering the environmental benefits of soil conservation, the two main control measures employed against GLS are the use of fungicides and deployment of maize hybrids with resistance to the disease [6,7].

GLS disease is characterized by the formation of rectangular lesions on maize leaves, which reduce the photosynthetic potential and ultimately yield of the crop [8]. GLS disease symptoms develop from the lower leaves upwards on a maize plant, reaching greatest intensity after flowering. Assessment of GLS disease on maize therefore often takes into account the progression of GLS lesions on a plant as well as the number and size of lesions [6,8].

Two species of Cercospora, which belong to the Dothidiomycetes family of fungi characterized by many foliar plant pathogens, are the causal agents of GLS on maize. *Cercospora zeae-maydis* and *Cercospora zeina* were first categorized as two types of *C. zeae-maydis*[9], however they have now been classified into separate species [10,11]. GLS lesions formed by each species appear to be identical, although growth *in vitro* and disease development is slower in *C. zeina*[9].

Differences in geographical distribution of the two species have been noted. *C. zeae-maydis* is the predominant pathogen in the USA, however *C. zeina* is found in the Eastern corn belt [9] and there are some regions where the ranges overlap, such as North Carolina [12]. GLS is found on maize throughout sub-Saharan Africa, and *C. zeina* appears to be the causal agent in Africa, with isolates from Zambia, Zimbabwe, Kenya, Rwanda, Uganda and South Africa being classified into the *C. zeina* group when subjected to molecular analysis [10,13,14].

The identification and characterization of maize germplasm with resistance to GLS has been an ongoing goal of maize researchers. Published work indicates that maize resistance to GLS is quantitative in nature, with additive gene action being commonly reported [15-17]. No cases of single gene qualitative resistance to GLS have been reported, and isolates of the pathogen have not been classified into races based on host specificity. However, since the molecular basis of resistance to GLS has not been characterized yet, it still remains possible that quantitative resistance could be effected by so-called weak resistance (R) genes that recognize pathogen effectors in a gene-for-gene manner [18]. A mechanism for this could be tolerance mediated through an R gene, as was recently shown in Arabidopsis pathosystems [19,20].

Quantitative trait loci (QTL) for resistance to GLS have been reported from several studies using maize populations developed from bi-parental crosses of inbred lines, where one parent showed greater GLS resistance compared to the other. Initial studies in the USA prior to 2008 employed F2, F2:3 or backcross populations to identify QTL, which limited the number of environments (field sites and seasons) that each population could be tested in [21-24]. Population sizes were between 100–200 and relatively low numbers of molecular markers such as RFLP were used. However, QTL were identified on all ten chromosomes of maize, although they were defined to regions of up to 30 cM (centiMorgans).

Subsequent studies employed populations of recombinant inbred lines (RIL), such as the intermated B73 X Mo17 (IBM) population, which allowed repeated GLS disease testing in multiple environments coupled with high density molecular marker maps [25-27]. QTL for GLS resistance were located to regions down to 3 cM [25]. Association mapping in a panel of 253 diverse inbred maize lines led to the identification of SNP polymorphisms in a glutathione *S*-transferase gene that were correlated with resistance to GLS [28].

Breeding programmes outside the USA have also led to the identification of QTL for GLS resistance. For example a South African inbred line V0613Y, first thought to carry a single major gene for resistance [29], was shown in parallel field trials at Cedara, KwaZulu-Natal, South Africa and Ohio, USA, to carry two QTL for GLS resistance [24]. The QTL on chromosome four was validated in progeny of a cross with a different susceptible parent [7]. In a separate study, two proprietary lines developed in southern Africa were used to detect different QTL for GLS resistance (on chromosome one and five) when a backcross population was tested at Hillcrest, a nearby site in KwaZulu-Natal, South Africa [30].

QTL for GLS disease resistance have been reported from field testing in Brazil of two different proprietary maize populations [3,31]. QTL mapping for GLS resistance has also been carried out in China [4]. A GLS resistance source from a breeding programme in Thailand was used. QTL on chromosomes five and eight were identified in repeated environments, and the QTL on chromosome eight was fine mapped to a region that corresponds to 1.5 Mb on the B73 genome [4]. The field trials were conducted in Yunnan province of China, where *C. zeina* and not *C. zeae-maydis* was isolated from all 25 locations sampled [32].

QTL for resistance to gray leaf spot disease have not been reported to be specific to either *C. zeae-maydis* or *C. zeina*[12], however it is useful to test for QTL in environments where one or other of the species is present. In South Africa, GLS is most prevalent in the subtropical environment of KwaZulu-Natal, and we recently showed that only *C. zeina* was isolated from multiple sites in this region [10]. We wished to identify sources of resistance to GLS caused by *C. zeina* in germplasm that had been developed under subtropical conditions in Africa, which could be taken up relatively quickly in local breeding programmes or deployed in hybrid combinations [16]. RIL populations have the advantage of being “immortal” so replicated trials can be planted in multiple sites and seasons, which mitigates the problem that GLS QTL identification can be variable from season to season due to differences in environmental conditions that affect disease severity [21,22].

The CML444 X SC Malawi RIL population was chosen for this study and planted at multiple sites in KwaZulu-Natal over a period of three years, resulting in the identification of seven QTL for GLS resistance. Two of these QTL corresponded to “hotspots” for the trait reported in previous studies on chromosome four and seven. In contrast, some QTL identified in this study have not been reported before, including one QTL on chromosome six that was identified in seven environments. This study represents one of the most comprehensive analyses of QTL for GLS due to the many field trials employed, and has a further advantage in defining QTL in response to *C.zeina*, since only this species is currently known to cause GLS in KwaZulu-Natal, South Africa.

## Methods

### Germplasm and field trials

A recombinant inbred line population (RIL, F7:S6) derived from a cross between subtropical white dent inbred lines CML444 and SC Malawi was used [33]. A total of 145 RILs, that produced sufficient seed and were phenotypically homogenous among plants of a RIL, were planted at five locations over three summer rainfall seasons (2008, 2009 and 2010) in KwaZulu-Natal, South Africa. Field trials were situated at Baynesfield Estate, Cedara Agricultural College, Hildesheim Farm, Redgates Farm and Ukulinga Research Farm of the University of KwaZulu-Natal. These field trial sites are within a 100 km radius from the city of Pietermaritzburg. All field sites were exposed to natural infection by *C. zeina*, and in addition, V5-V7 stage plants were inoculated in the whorls with dried and powdered material from the previous season’s GLS diseased maize plants. GLS disease severity data was recorded for all three seasons at Redgates Farm, two out of the three seasons at Cedara Agricultural College, Baynesfield Estate and Ukulinga Research Farm, and a single season at Hildesheim Farm. An “environment” was defined as a field trial with a particular planting date at a particular location.

For each trial, the RILs were planted in a randomized block with two or three replicates. Each replicate of a RIL was a row of 10 plants. GLS disease severity was scored on a per row basis using a 1–9 scale, where 1 and 9 represent no GLS disease and complete GLS susceptibility, respectively [6]. GLS disease scores were taken twice each season before and after anthesis, and a GLS disease score for each replicate row was obtained by averaging the scores from the time points.

Days to anthesis (DTA) was measured as days from planting to anthesis (pollen release) in 50% of plants in a row at Redgates Farm in the 2010 season. This field trial was distinct from those used for collection of GLS severity data since this represented a control plot sprayed with a fungicide regime and therefore no GLS symptoms were present (data not shown).

### Statistical analysis of field trial data

GLS disease scores were analyzed using the analysis of variance (ANOVA) function of the *car* package in R statistical software [34,35] to estimate the variance components attributable to environment (field trials), replicates within an environment, genotype and genotype × environment interactions. The PROC GLM procedure of SAS 9.3 (SAS Institute, Cary, NC) was used to calculate least square means of the GLS disease scores for each field trial using a mixed model approach considering replication as random effects and genotypes as fixed effects. These least square mean values were used to perform QTL mapping of the GLS disease phenotype for each field trial. In addition, “joint” GLS QTL for all environments (trials/seasons) were determined by (i) computing the z-score of the least square means of GLS disease scores for each RIL per environment; and (ii) using the mean z-score per RIL across all environments as input data for QTL mapping. Correlations between GLS disease phenotypes in different field trials were calculated using GraphPad Prism 5.04 (GraphPad Software Inc.).

### Genetic map enrichment

The genetic map of the CML444 X SC Malawi population which was previously constructed with 79 RFLP and 81 SSR markers, [33] was scrutinized to identify regions where there were gaps of approximately 20 cM or more. SSR markers in these regions were selected from the Maize Genetics and Genomics database (http://www.maizegdb.org/). DNA was extracted from a pool of three plants per RIL for 145 RILs, and genotyped by polyacrylamide or agarose gel electrophoresis with the following SSR markers: bnlg1811, bnlg615, umc1111, phi073, bnlg1449, bnlg1108, umc1720, bnlg105, dupssr10, umc1155, umc1572, bnlg2191, umc1413, umc1424, umc1562, umc1170, bnlg1375, umc1137, umc1337. Genetic map construction was carried out using the genetic map data from Messmer et al. [33] plus the additional SSR marker genotypes using MapManager QTX software [36]. As the mapping population consisted of 145 RILs, confidence in markers which were closer than 5 cM to another marker was low and therefore those markers were removed to reduce possible distortion of the map. The final genetic map, named QMap 2.0, was displayed using MapChart [37].

### QTL analysis

QTL for GLS disease severity as well as DTA in the CML444 X SC Malawi RIL population were identified for each field trial based on the genetic map QMap 2.0 and applying the Composite Interval Mapping (CIM) utility in Windows QTL Cartographer 2.5_011 [38] using the standard model 6 with a window size of 10 cM and a 1 cM walk speed. Both forward and backward regression analysis was performed. The statistical significance LOD (logarithm of odds) threshold used to declare the presence of QTLs was obtained from 1000 permutations at a genome-wide significance level of 5% for each field trial [39,40]. The 1- and 2-LOD support intervals were used to define each QTL region. Epistatic interactions between QTL were assessed by using the Multiple Interval Mapping (MIM) utility in Windows QTL Cartographer as previously described [41].

### QTL comparison

The maize core bin positions of QTL for GLS from literature were retrieved to compare with consensus QTL identified in the current study. Most publications designate GLS QTL into maize core bins [3,4,7,25-27]. A maize genome bin has been defined as one of the 100 designated chromosomal segments between two core RFLP markers (http://www.maizegdb.org/cgi-bin/bin_viewer.cgi). The positions of these bin markers have been placed onto high density maize maps, such as the IBM2 2005 neighbours frame map (3287 well-ordered markers), as well as the B73 genome sequence which enables most studied QTLs to be placed into maize core bins based on common markers [42]. The maize bin positions of GLS QTL from five studies prior to 2008 [21-24,30] were taken from the analysis by Balint-Kurti et al. [25]. The study of Juliatti et al. [31] could not be included in the comparison as there was insufficient information to place the proprietary SSR markers on the IBM2 2005 map.

## Results

### Gray leaf spot disease assessment of maize RIL population

**Figure 1:**
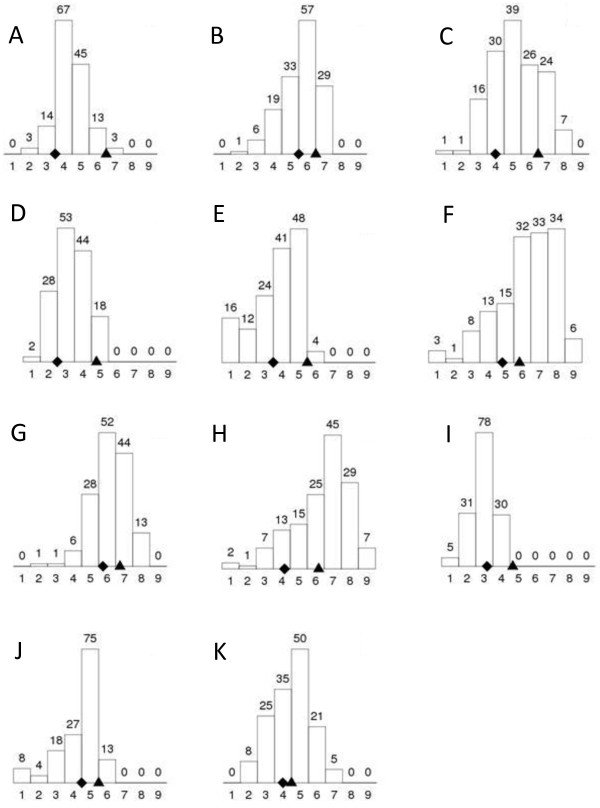
**Distribution of average GLS disease scores in the 145 RILs of the CML444 X SC Malawi population in each of the environments.** The GLS disease scores on a 1-9 scale from resistant to susceptible are shown on the x-axis, and the number of RILs with each disease score is plotted on the y-axis and written above each column. The GLS disease scores for CML444 (♦) and SC Malawi (▲) are shown on the x-axis. The environments were: **A-C**, Redgates 2008, 2009, 2010, respectively; **D-F**, Ukalinga 2008 (planted 29 Nov 2007), Ukalinga 2008 (planted 19 Dec 2007), Ukalinga 2009, respectively; **G**, Hildesheim 2009; **H-I**, Baynesfield 2009, 2010, respectively; **J-K** Cedara 2008, 2009, respectively.

Typical GLS disease symptoms were observed at all five locations where the CML444 X SC Malawi RIL population was planted in KwaZulu-Natal, South Africa. GLS disease data was collected from eleven environments over the three year period (Figure 1). *C. zeina* has recently been shown to be the causal agent of grey leaf spot disease in this region [10]. The parental line SC Malawi (average GLS score across trials = 5.2) was more susceptible to GLS than the parental line CML444 (average GLS score = 3.5) in all environments (Figure 1). Transgressive segregation was observed as many RILs were more susceptible (highest average GLS score = 6.3) or more resistant (lowest average GLS score = 2.0) than the parents (Figure 1). Most environments showed a wide distribution of disease scores, consistent with quantitative and not qualitative resistance (Figure 1). Correlation between environments ranged from 0.39 to 0.85 with a median of 0.67 (data not shown). Cedara is a well-known hotspot of GLS disease [2,24] and GLS disease expression was good (Figure 1J & K), as was the case at Redgates in all three seasons (2008; 2009; 2010; Figure 1A-C). Both plantings of the RIL population at Ukalinga in the 2008 season had lower GLS disease levels (Figure 1D & E). This site is not known for high GLS disease levels (P.Tongoona, pers. comm.), however GLS disease levels were higher in 2009 (Figure 1F). The 2009 season produced higher GLS disease levels at Baynesfield and Hildesheim, in contrast to 2010 at Baynesfield, where GLS disease was lower on account of the low rainfall in February/March 2010, prior to anthesis (Figure 1).

GLS disease phenotypes were analyzed using ANOVA (Table 1). The environment, replication within environment, genotype as well as the genotype × environment interactions were significant (*P* < 0.001) contributors to phenotypic variance observed in the GLS disease scores across the eleven environments. The contribution to variation due to differences between environments was considerably larger than the variation attributed to genotype, replication within environment and genotype × environment (Table 1). As there was a significant genotype × environment interaction effect, QTL analyses were conducted separately for each of the eleven field trials. The high level of environmental variation observed could be ascribed to seasonal variation between 2008, 2009 and 2010 as well as factors like humidity, temperature and rainfall between the different field trail locations. These factors could influence the amount of fungal sporulation thereby affecting efficiency of infection by *C. zeina*.

**Table 1:** Analysis of variance of gray leaf spot (GLS) disease scores from a population consisting of 145 RILs from a CML444 X SC Malawi cross scored over eleven environments in KwaZulu-Natal, South Africa

**Source**	**d.f.**^**b**^	**SS**^**c**^	**MS**^**d**^	**F-value**	***P*-value**
Environment^a^	10	5142	514	101	< 0.001*
Replications within environment	2	209	11	12	< 0.001*
Genotype	144	3972	28	31	< 0.001*
Genotype x environment	1437	2052	1.4	1.6	< 0.001*
Residuals	2727	2413	0.9		

^a^The RILs were planted in a randomized block with two or three replicates for each environment.

^b^Degree of freedom.

^c^Sum of squares.

^d^Mean square.

*Significant at *P* < 0.001.

### Genetic map construction

**Figure 2:**
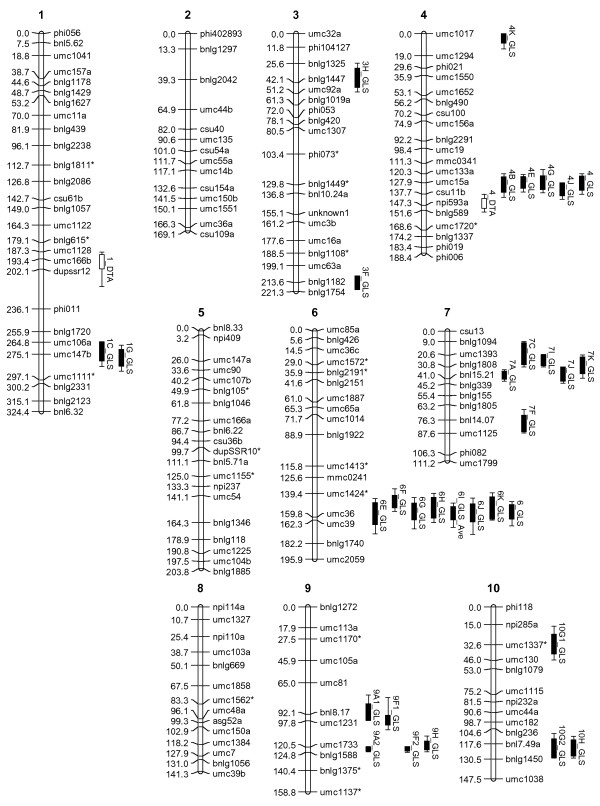
**Q-Map 2.0 - Genetic linkage map (167 markers) from the RIL population (n = 145) derived from the parental lines CML 444 and SC Malawi.** Each marker position (cM) and marker name are shown on the left and right of each chromosome, respectively. Markers added to the map obtained from Messmer *et al.*[33] are indicated by *. QTL for days to anthesis (white bars), GLS resistance per field trial (black bars) and joint QTL for GLS resistance (cross hatch bars) are shown on the right of relevant chromosomes (1-LOD intervals i.e. 95% confidence intervals). The 2-LOD intervals (99% confidence intervals) for each QTL are indicated by lines on each side of the 1-LOD intervals.

The genetic linkage map for this CML444 X SC Malawi RIL population reported in Messmer et al. [33] was enriched by mapping of an additional 19 SSR markers to fill gaps greater than ~20 cM. The resultant map was called QMap 2.0 and was made up of 167 markers based on data for 145 RILs with a total map size of 1862 cM (Kosambi) (Figure 2).

### Identification of QTL for GLS resistance

Composite interval mapping of GLS disease severity data of the CML444 X SC Malawi RIL population from each of the eleven environments (over three seasons and five field locations) based on QMap2.0 identified 30 QTL for GLS resistance in at least one environment in KwaZulu-Natal, South Africa (Table 2, Figure 2). Twenty of the QTL had R^2^ values greater than 10%, which is an estimate of the phenotypic variation explained by the QTL. Some GLS QTL were identified in multiple environments, for example the QTL on chromosome 4 and chromosome 6, which were both identified as joint GLS QTL when data from all eleven environments was combined for QTL mapping (Table 2, Figure 2). These two QTL were identified together in three environments (Ukalinga (late planting 2008), Hildesheim (2009) and Cedara (2008) (E, G, J on Table 2, respectively)). Most of the QTL were identified in at least two environments. No particular field site was superior for QTL identification: six QTL each were identified at Redgates, Baynesfield and Cedara, whereas seven QTL were identified at Ukalinga and five QTL at Hildesheim (Table 2). GLS QTL are known to be highly environment dependent, with hot and humid conditions prior to anthesis being important for disease development [5,24]. The importance of timing was illustrated by the fact that no QTL was identified from the early planting at Ukalinga in 2008, whereas two QTL were identified from the trial planted at the same site but three weeks later (Table 2). The lack of repeated identification of the same QTL at the same site over different seasons is most likely due to the varying climate conditions from season to season.

**Table 2:** **QTL for gray leaf spot (GLS) resistance and days to anthesis (DTA) identified in the CML444 X SC Malawi RIL population**^$^

**Chr**^**a**^	**Bin**^**b**^	**Peak marker**^**c**^	**1-LOD interval**^**d**^	**2-LOD interval**^**e**^	**LOD Score**^**f**^	**R**^**2 g**^	**Additive effect**^**h**^	**Allele source**^**i**^	**QTL name**^**j**^
1	1.10	umc147b	264.7 - 281.0	264.3 - 285.7	3.39	10.1	-0.460	CML	1C_GLS
1	1.10	umc147b	270.9 - 285.6	267.5 - 289.7	3.23	6.4	-0.278	CML	1G_GLS
3	3.02/3.03	bnlg1447	29.6 - 46.2	25.3 - 50.1	2.95	8.5	-0.409	CML	3H_GLS
3	3.09	bnlg1182	201.8 - 219.6	207.9 - 219.6	4.55	13.0	-0.472	CML	3F_GLS
4	4.01	umc1017	0.0 - 8.2	0.0 - 13.3	3.14	8.0	-0.252	CML	4K_GLS
4	4.08	umc15a	121.9 - 133.8	116.5 - 136.6	4.78	10.9	-0.364	CML	4G_GLS
4	4.08	umc15a	122.6 - 132.9	120.9 - 136.0	4.55	12.8	-0.551	CML	4E_GLS
4	4.08	umc15a	122.9 - 136.1	120.4 - 140.5	3.59	8.9	-0.336	CML	4B_GLS
4	4.08	csu11b	127.9 - 139.3	127.9 - 142.3	3.92	12.0	-0.497	CML	4J_GLS
6	6.06	umc1424	141.0 - 151.7	135.3 - 154.7	4.92	21.8	0.626	SC	6F_GLS
6	6.06/6.07	umc36	142.7 - 160.5	139.2 - 164.3	3.29	13.0	0.504	SC	6H_GLS
6	6.06/6.07	umc36	142.3 - 161.4	139.0 - 162.3	2.95	13.8	0.335	SC	6K_GLS
6	6.06/6.07	umc36	147.3 - 166.1	143.7 - 174.0	3.64	13.7	0.584	SC	6E_GLS
6	6.06/6.07	umc36	147.5 - 162.1	143.1 - 169.6	5.00	10.2	0.362	SC	6G_GLS
6	6.06/6.07	umc36	150.7 - 162.0	147.7 - 168.5	4.61	10.7	0.232	SC	6I_GLS
6	6.06/6.07	umc36	148.3 - 163.9	143.2 - 174.4	3.38	9.3	0.451	SC	6J_GLS
7	7.02	umc1393	10.0 - 28.7	9.0 - 30.7	3.16	10.2	0.467	SC	7C_GLS
7	7.02	umc1393	20.4 - 29.7	20.1 - 30.8	3.31	10.4	0.218	SC	7I_GLS
7	7.02/7.03	bnlg1808	22.1 - 36.5	20.4 - 40.5	3.00	9.0	0.273	SC	7K_GLS
7	7.02/7.03	bnlg1808	34.4 - 41.1	33.0 - 43.0	4.67	13.4	0.341	SC	7A_GLS
7	7.02/7.03	bnl15.21	30.8 - 42.8	30.8 - 44.6	3.16	9.4	0.446	SC	7J_GLS
7	7.04	bnl14.07	72.2 - 86.3	67.2 - 87.4	3.26	10.0	0.425	SC	7F_GLS
9	9.04/9.05	umc1231	83.0 - 97.3	75.6 - 97.6	3.31	10.7	-0.317	CML	9A1_GLS
9	9.04/9.05	umc1231	92.9 - 101.3	77.8 - 105.5	3.70	9.2	-0.435	CML	9F1_GLS
9	9.06	umc1733	115.0 - 122.8	110.7 - 124.5	3.40	7.6	0.398	SC	9H_GLS
9	9.06	umc1733	119.8 - 123.6	119.8 - 124.7	4.51	10.1	0.482	SC	9F2_GLS
9	9.06	umc1733	120.3 - 123.6	120.0 - 124.4	6.65	17.8	0.443	SC	9A2_GLS
10	10.2	umc1337	23.4 - 40.5	16.6 - 45.5	2.99	6.3	-0.283	CML	10G1_GLS
10	10.06/10.07	bnl7.49a	114.0 - 127.9	110.9 - 129.8	4.53	13.5	-0.509	CML	10H_GLS
10	10.06/10.07	bnl7.49a	113.4 - 129.2	108.7 - 129.9	4.52	12.5	-0.391	CML	10G2_GLS
4	4.08	umc15a	122.3 - 134.4	120.4 - 138.0	3.90	10.6	-0.275	CML	4_GLS*
6	6.06/6.07	umc36	148.8 - 161.4	146.2 - 167.2	6.09	20.9	0.392	SC	6_GLS*
1	1.07	dupssr12	189.7 - 202.0	187.5 - 217.2	4.73	14	1.1399	SC	1_DTA^#^
4	4.09	npi593a	141.6 - 149.8	138.0 - 153.1	4.04	10	0.9455	SC	4_DTA^#^

^a^Maize chromosome.

^b^Chromosome bin location of QTL (1-LOD (i.e. 95% confidence) interval) based on shared markers with the IBM2005 neighbours frame map.

^c^Peak marker refers to marker on QMap 2.0 that is closest to the QTL peak.

^d^Range in cM that defines 1-LOD interval of QTL.

^e^Range in cM that defines 2-LOD interval of QTL.

^f^Log of odds (LOD) value at position of QTL peak.

^g^Phenotypic variance explained by the QTL (expressed as percentage).

^h^Additive effect of QTL. For GLS disease ratings, this is based on the one to nine scale employed. For days to anthesis, this is based on days. Positive values indicate that the allele for resistance or early anthesis was derived from SC Malawi.

^i^Parental allele associated with increased GLS resistance or earlier anthesis CML = CML444; SC = SC Malawi.

^j^QTL name. First number indicates maize chromosome number whereas letter denotes field trial as described in Figure 1. Subsequent numbers differentiate more than one QTL identified on the same chromosme in the same field trial. GLS represent QTL for GLS resistance whereas DTA indicate QTL for days to anthesis.

*Joint GLS QTL calculated from the mean z-scores of the least square means of the GLS disease scores from the eleven environments.

^#^DTA QTL were identified using DTA data from an independent field trial at Redgates 2010.

^$^GLS QTL were identified using QMap2.0 and GLS disease data from each of eleven environments in KwaZulu-Natal, South Africa. DTA QTL were identified using QMap2.0 and DTA data from an independent field trial at Redgates farm, KwaZulu-Natal, South Africa.

The thirty QTL identified in eleven environments were consolidated into seven QTL that were detected in two or more environments and were placed in the same maize core bins based on common markers with the IBM2 2005 map (QTL in bin 1.10, 4.08, 6.06/6.07, 7.02/7.03, 9.04/9.05, 9.06, 10.06/10.07) plus six remaining QTL that were identified in one environment each (Table 2).

### Comparison of QTL with previously identified QTL for GLS resistance

The CML444 X SC Malawi QTL identified in this study were compared with GLS resistance QTL identified in eleven previous studies based on the maize core bin positions of the QTL (Table 2) (Additional file 1).

Several of the GLS resistance QTL identified in the CML444 X SC Malawi population cluster with QTL found in previous studies (Additional file 1). The QTL on chromosome 1 (bin 1.10) overlaps with a QTL reported by Zhang et al. [4]. The QTL with the allele associated with GLS resistance derived from CML444 in bin 4.08 was identified in four environments as well as the joint GLS QTL (Table 2, Additional file 1). Both inbred line VP31 and its progenitor VO163Y have a QTL in this bin [7,24], which also overlap with QTL reported by Bubeck et al. [21] and Saghai Maroof et al. [22]. The GLS QTL with the SC Malawi allele associated with resistance in bin 7.02/7.03 overlaps with QTL from four studies [3,21,23,26] (Additional file 1). QTL with the resistance alleles from CML444 in bins 9.05 and 10.06 overlap with QTL from B73 and B73rhm, respectively [21,25].

There were also several QTL for GLS resistance from the CML444 X SC Malawi population that have QTL that do not overlap with QTL reported in previous studies, notably (i) the bin 6.06/6.07 QTL with the SC Malawi allele associated with resistance that accounted for 9.3- 21.8% of the phenotypic variation in seven environments and as a joint GLS QTL; and (ii) the bin 9.06 QTL with the SC Malawi allele associated with resistance identified in three environments (Table 2, Additional file 1). Single environment GLS QTL not previously reported were identified in bin 3.02/3.03, bin 3.09, bin 4.01, bin 7.04, and bin 10.2 (Table 2).

## Discussion

QTL mapping of the CML444 X SC Malawi population in eleven environments in KwaZulu-Natal, South Africa, over three years revealed seven QTL for GLS disease resistance that were observed in at least two environments (GLS QTL in bin 1.10, bin 4.08, bin 6.06/6.07, bin 7.02/7.03, bin 9.04/9.05, bin 9.06, bin 10.06/10.07; Table 2). Detection of QTL for GLS resistance has been reported to be variable from site to site and season to season [21]. For example, Saghai Maroof et al. [22] identified three QTL that were consistent in F2 and F3 generations and seasons, but another two QTL that were not. The use of a RIL population in our study allowed testing in multiple environments, which gave confidence in the detected QTL. Furthermore, the QTL were identified using an improved genetic map compared to the map reported by [33], since we included 19 additional gap-filling SSR markers (Figure 2).

Prior to our study, there were two reports of QTL for GLS resistance that were identified under field conditions in South Africa. Lehmensiek et al. [30] used a bi-parental backcross population from a proprietary source, and identified GLS resistance QTL on chromosome one (bin 1.05/1.06) and chromosome five (bin 5.03-5.06). In another study, QTL for GLS resistance in the inbred line V0613Y were mapped to chromosome four (bin 4.06/4.08) and chromosome two (bin 2.09) [24]. The source of GLS resistance in this inbred line was proposed to be either a terminal-ear maize type or teosinte germplasm developed in a breeding programme in South Africa [29]. These studies were carried out at field sites in KwaZulu-Natal that are within a 100 km radius of Pietermaritzburg and likely to have similar GLS disease pressure, although the diversity and identity of the Cercospora species was not known at the time. In the current study, we identified QTL in a different population derived from subtropical germplasm. SC Malawi was developed in Zimbabwe in the 1960s, whereas CML444 was developed by CIMMYT breeding programmes in Mexico and Zimbabwe [33]. Consensus QTL from our study did not overlap with those reported by Lehmensiek et al. [30], whereas we did detect a QTL that coincided with the QTL on chromosome four from V0613Y (Additional file 1). This may reflect the uptake of antecedents of V0613Y in breeding programmes in southern Africa.

Some of the GLS resistance QTL identified from the CML444 X SC Malawi population in our study corresponded to clusters or “hotspots” of GLS resistance QTL reported in other populations (Additional file 1). Despite the limitations of comparing QTL positions between different studies which used different genetic maps, population sizes, and statistical tests to define QTL, it has proven useful to compare QTL based on a common framework of maize core bin regions [25,42]. Previous identification of hotspots of GLS QTL were carried out prior to 2007 and included six populations [28,43]. Visual inspection of the data of Wisser et al. [42] for QTL reported in at least three of the six populations revealed GLS QTL hotspots in bin 1.05/1.06, bin 2.05/2.06, bin 4.08, and bins 5.03 and 5.05 [42]. These correspond relatively well with “consensus QTLs” defined by Shi et al. [43] who also projected QTL from the same studies onto the IBM2005 map, and noted seven “consensus QTLs” (bin 1.06, bin 2.06, bin 3.04, bin 4.06 (two), 4.08, 5.03 and 8.06).

Our study was able to extend the analyses of Wisser et al. [42] and Shi et al. [43], since there have been seven subsequent studies with different germplasm sources including our own (Additional file 1). We defined QTL hotspots as bins with QTL in four or more of thirteen populations, namely bin 1.05/1.06, bin 2.03/2.04, bin 4.08, bin 5.03/5.04 and bin 7.02/7.03 (Additional file 1). Our analysis confirms the previous demarcation of GLS QTL hotspots, and adds the hotspot on chromosome seven. The CML444 X SC Malawi population did not show QTL overlapping the bin 1.05/1.06 or bin 5.03/5.04 QTL hotspots, but did overlap with bin 4.08 and bin 7.02/7.03 hotspots.

Hotspot analysis by comparing QTLs from different studies has value in identifying common genomic regions across a germplasm collection with a significant effect on a trait of interest. For example, a hotspot may correspond to a common GLS resistance allele or may be a region that has multiple alleles/genes that can confer GLS resistance. Candidates for the latter are R genes, which are often found in clusters [44]. Wisser et al. [42] pointed out R genes in GLS resistance hotspots in bin 1.05/1.06, bin 4.08 and bin 5.03. However, hotspot analysis has some caveats. For example, a hotspot might be observed if a particular inbred line with rare alleles is used as a parent in a large proportion of the populations under study. This may be the case for the inbred line B73. Common alleles may also be hidden in QTL mapping studies when they are present in both parents of most populations, since the alleles would appear to be rare.

Different breeding programmes have produced germplasm with GLS resistance QTL in the same QTL hotspot. For example, the hotspot on chromosome one has been reported from populations with diverse origins [3,22,23,30], including a Mo17 allele (bin 1.05) [25]. This may represent a cluster of R genes or alleles. The QTL in bins 4.06/4.08 from line V0613Y from South Africa [24], which was mapped to bin 4.08 in a progeny line VP31 [7], overlaps with a QTL from the ADENT inbred line [21]. V0613Y and ADENT were developed in different breeding programmes on different continents [21,29]. Interestingly, B73 carries a susceptible allele in this bin region in the ADENT X B73rhm population, whereas it confers a resistant allele in the Val14 X B73 population [21,22].

The QTL in bin 4.05 appears to contain alleles for GLS resistance from different sources. Balint-Kurti et al. [25] report a QTL from B73 that was only observed in the IBM population, and not the “Stuber” population. They propose that although these populations have the same parents, there are opposing effects at closely linked loci that cancel out the QTL in the “Stuber” population which has fewer recombination events. Interestingly, Zwonitzer et al. [26] identified a GLS resistance allele from line Ki14 and not the other parent B73 in this bin (Additional file 1). There is a similar case in bin 8.05, where there is a resistant allele from B73 in the B73 X ADENT population [21], but a CML52 allele conferring resistance from the B73 X CML52 population [27].

From the above results from different inbred lines, there is substantial evidence for allelic series at QTL loci for GLS resistance and susceptibility, as have been reported for studies with NAM populations for other maize diseases such as southern corn leaf blight [45].

Two independent studies [4,26] identified different alleles for GLS resistance when they used different parents from the Suwan-1 population released in 1975 by Kasetsart University in Thailand, indicating that there are multiple sources of resistance in this population. Zwonitzer et al. [26] reported GLS resistance alleles from line Ki14 (derived from the Suwan-1 population) on chromosomes four, seven and ten, whereas Zhang et al. [4] did not report GLS resistance alleles on these chromosomes, but detected GLS resistance alleles from line Y32 (derived from the Suwan-1 population) on chromosome five and eight not present in the former study (Additional file 1).

The proprietary germplasm of Pozar et al. [3] has GLS resistance alleles that overlap with QTL in bin 1.05/1.06 from line 061 [23], as well as QTL in bin 7.02/7.03 reported from this line and line NC250A [21] (Additional file 1). This may indicate that these lines were used in the development of the proprietory parental lines [3].

There appears to be a relative paucity of GLS resistance QTL on chromosomes three, six, eight, nine and ten (Additional file 1). QTL identified in the CML444 X SC Malawi population that are most likely to be unique were the GLS resistance QTL in bins 6.06/6.07 and 9.06 (Table 2, Additional file 1). The QTL in bin 6.06/6.07 was identified in seven environments and explained on average 13% of the variation in the data, with the resistance allele derived from SC Malawi (Table 2). The QTL in bin 9.06, with resistance also derived from the more susceptible parent, explained 12% of the phenotypic variation. There were no significant epistatic effects between any pairwise combination of QTL (data not shown), as was observed in a GLS QTL study of another RIL population [25].

Previous authors have observed that there may a link between flowering time and GLS resistance with later maturing plants showing more resistance [21,23,25]. CML444 is a later maturing genotype than SC Malawi as reported in [33], and this was confirmed in the current study in KwaZulu-Natal, where DTA for CML444 was on average five days later than SC Malawi (data not shown). Interestingly, QTL for DTA measured in this study in bins 1.07 and 4.09 (Table 2) corresponded to flowering time QTLs reported for field trials in Mexico and Zimbabwe in [33]. However, these QTL did not overlap with GLS resistance QTLs identified in this study, although the DTA QTL in bin 4.09 is close to the main effect GLS QTL in bin 4.08 observed in multiple environments (Figure 1, Table 2).

*C. zeina* and not *C. zeae-maydis* was the pathogen causing GLS in KwaZulu-Natal during the course of this study of the CML444 X SC Malawi population ([10] and data not shown). Although there are no reports of differential response in maize lines to these two species of fungus [12], we can conclude that the QTL observed in this population are effective against *C. zeina* prevalent in South Africa. The only other study where the causal agent is most likely to be *C. zeina* exclusively is that of Zhang et al. [4] in Yunnan province of China [32]. It would be interesting to test whether the QTL identified in the CML444 X SC Malawi population are also identified in environments where *C. zeae-maydis* is the causal agent of GLS. This would be an important consideration for breeding initiatives in different countries. Future work in our group will address this question by conducting field trials of the same maize RIL populations in paired environments where only *C. zeae-maydis* or only *C. zeina* are found. Species-specific PCR diagnostic assays developed in our group that can be applied directly from GLS lesions will be used to confirm the prevalent species of fungus in each environment [8,10].

## Conclusions

QTL mapping of the CML444 X SC Malawi RIL population at five sites over three years in KwaZulu-Natal, South Africa allowed the identification of seven QTLs for resistance to GLS caused by *C. zeina*. The seven QTL were observed in at least two environments and were positioned in common bins in the maize genome. Although analysis of variance indicated that environment had the greatest effect on phenotypic variation, the study succeeded in identifying GLS QTL at high confidence since data was collected from a large number of field trials. Comparison of the QTL from this study with QTL reported from previous studies indicated that QTL from the CML444 X SC Malawi population in bin 4.08 and bin 7.02/7.03 coincided with hotspots of GLS QTL in the maize genome. The QTL in bin 4.08 has previously been reported from the inbred line V0613Y from South Africa [24]. Two novel QTL (bin 6.06/6.07 and 9.06) were discovered in this study, which interestingly had alleles for increased resistance to GLS derived from the susceptible parent SC Malawi.

## Competing interests

The authors declare no competing interests.

## Authors’ contributions

PT, FM and FK designed and conducted the field experiments, assessed GLS disease, and analyzed the data. JK constructed the genetic map and contributed to the analysis. MC performed much of the data analysis. DB and AM contributed to the design, analysis and interpretation of the data. DB conceived the study, secured the grants, and wrote the manuscript. All authors read and approved the final manuscript.

## Supplementary Material

Additional file 1 Comparison of maize core bin positions of QTL for GLS resistance identified in at least two environments from the CML444 X SC Malawi RIL population with QTL from eleven other studies. Click here for file
